# Incidence, Size and Orientation of Maxillary Sinus Septa—A Retrospective Clinical Study

**DOI:** 10.3390/jcm11092393

**Published:** 2022-04-24

**Authors:** Laura Andreea Schiller, Horia Mihail Barbu, Stefania Andrada Iancu, Silviu Brad

**Affiliations:** 1 Department of Radiology, Victor Babes University of Medicine and Pharmacy, 300041 Timisoara, Romania; dr.grosulaura@gmail.com (L.A.S.); bradsilviu@yahoo.com (S.B.); 2 Department of Oral Surgery, Stomdas Clinic, 600125 Bacau, Romania; 3 Oral Implantology Department, Faculty of Dental Medicine, Titu Maiorescu University, 031593 Bucharest, Romania; horia.barbu@gmail.com; 4 European Centre of Oral Implantology, 011473 Bucharest, Romania; 5 Department of Prosthodontics, Faculty of Dental Medicine, Titu Maiorescu University, 031593 Bucharest, Romania

**Keywords:** sinus septa, maxillary sinus, sinus floor augmentation, sinus septum classification

## Abstract

Background: The purpose of this study is to analyze if there is any statistical correlation between the surgery’s complexity (easy to difficult—depending on the anatomical conditions) and the patient’s sex, type of edentulism, and left or right side of the maxilla. Methods: Cone beam computed tomography records of 1192 maxillary sinuses were evaluated, measured, and statistically analyzed with respect to patient sex, type of edentulism, and left or right side, taking into consideration Wen’s proposed sinus septum classification. Results: Our research suggests that most sinus augmentation procedures in patients presenting antral septum fall into the Moderate A category (31.94%) and that there is not a correlation between the surgery’s complexity (easy to difficult) and the patient’s sex, type of edentulism and left or right side of the maxilla. Conclusion: We suggest a minor modification to Wen’s classification in view of the fact that our findings revealed a combination of medio-lateral and antero-posterior septa that we could not classify in one of the existing categories.

## 1. Introduction

Implant dentistry offers the possibility to rehabilitate simple and complex cases of edentulism with the aid of various, convenient materials and techniques. When necessary, implant surgeries for the maxilla are accompanied by sinus floor augmentation procedures to ensure the quantity and quality of bone in the posterior region of the dental arch.

Good knowledge of the anatomical variation of the maxillary sinus is mandatory for oral surgeries performed in immediate proximity (implant insertion in the subantral bone), or directly in the sinus cavity (sinus floor elevation).

The antral septa represent the most frequent disparity when describing the anatomy of the maxillary sinus [1]. These are cortical bone extensions arising from the floor or from the walls of the maxillary sinus [2,3].

The presence of sinus septa is associated with Schneiderian membrane perforation during sinus floor elevation, which increases surgical morbidity and enchains possible complications such as graft displacement, graft infection, sinusitis, etc.

A preoperative radiological assessment of the maxillary sinus provides important information about the presence of sinus septa, their location, size, and orientation. In this way, the surgeon can anticipate possible intraoperative complications and predict the complexity of the procedure. In some cases, the sinus septa hinder the luxation of the bony window, thus a modified design of the osteotomy window is needed as an alternative to access the sinus cavity [4]. This study aims to evaluate the incidence of maxillary sinus septa and to introduce a modification to Wen’s classification of the maxillary sinus septa [1]. Wen et al. divide the sinuses presenting septa into three categories based on location, number, orientation, and size of antral septa: easy (E)—subclass A and B, moderate (M)—subclass A and B, and difficult (D)—subclass A, B, and C. Corresponding treatment approaches were suggested for each category [1].

Different examination techniques for evaluation of the maxillary sinus include standard oral radiography, orthopantomography, CBCT scans [5], ultrasonography, and magnetic resonance imaging [6,7,8,9]. CBCT allows high-resolution images and does not have the inherent drawback of superimposition and magnification [5,10]. Although CBCT is considered the gold standard, the patient’s exposure to ionizing radiation and therefore biological damage must be constantly monitored. For this reason, the development and improvement of the applications of radiation-free diagnostic tests such as ultrasounds and MRI (Magnetic Resonance Imaging) in the study of the maxillary sinus, in particular, is a great stimulus for future research [6,7,8].

## 2. Materials and Methods

### 2.1. Study Design

The following research was structured as a retrospective cohort study, respecting the STROBE (Strengthening the Reporting of Observational Studies in Epidemiology) guidelines. Maxillary sinuses in dentate, single, partial, and edentulous patients were analyzed on CBCTs (cone beam computed tomographies) from the point of view of the bony internal architecture and the antral septa, respectively. The radiographic findings belonged to the European Center of Implantology, located in Bucharest, Romania, and the Central Clinic located in Bacau, Romania. All patients included in the study group agreed to be part of the study and signed an informed consent document.

The CBCTs were performed for these patients for accurate diagnosis and treatment plan in various medical circumstances (orthodontics, sinus lift procedures, third molar impaction, maxillary sinus pathologies, implant insertion, etc.) and were later introduced in this research. Unnecessary exposure for CBCT analysis was not performed. The research respected the ethical principles of the Helsinki Declaration (2008) and the additional revisions included at Fortaleza (2013), taking into consideration to protect the health and rights of the patients.

For this research to begin, ethical approval was firstly signed by the Institutional Review Board within STOMDAS CLINIC (Bacau, Romania) (No STO07FEB22-C01/7 February 2022).

### 2.2. Study Population

Medical documents of the patients treated in the two clinics were screened for potential inclusion in this research.

Inclusion criteria:

- Age > 18 years, with no other age or gender restrictions;
- Partial (hemimaxilla) or total CBCTs comprising at least 2/3 of the floor of the sinus cavity;
- CBCTs of the upper arch.

Exclusion criteria:

- CBCTs with exposure errors (because of patient’s movement, other artefacts);
- Maxillary sinus cavities with previous sinus grafts;
- CBCTs which do not comprise entirely, anteroposteriorly the sinus cavity;
- CBCTs with a field of view of 40 × 40 (for endodontic purposes);
- Absence of patient’s informed consent;
- Panoramic radiographies.

### 2.3. Radiographic Measurements

The radiological exams were performed by two different cone beam computed tomography apparatuses. One of the computed tomographs was Veravierpocs RD R100 CB (J. Morita Corporation, Tokyo, Japan) with 10 mA and 0.125 voxel size. The second device was Kavo OP 3D Pro (Kavo, Biberach an der Riss, Germany), having 18.54 mA and 0.25–0.4 voxel size. Image processing, as well as the measurements, were performed in One Volume Viewer (J. Morita Corporation, Tokyo, Japan) and OneDemand3D^TM^ X-ray software (Cybermed, Daejeon, Korea), accordingly.

The analysis of the internal architecture of the maxillary sinus was accomplished by two examiners, who evaluated the antrum in coronal, axial, and sagittal planes, while 3-dimensional reconstructions were used as necessary. To avoid any errors in the localization of septa, the multiplanar reconstruction technique was used [11]. The operators calibrated the software with a slice thickness of 0.5 mm and a slice interval of 1 mm.

**Figure 1:**
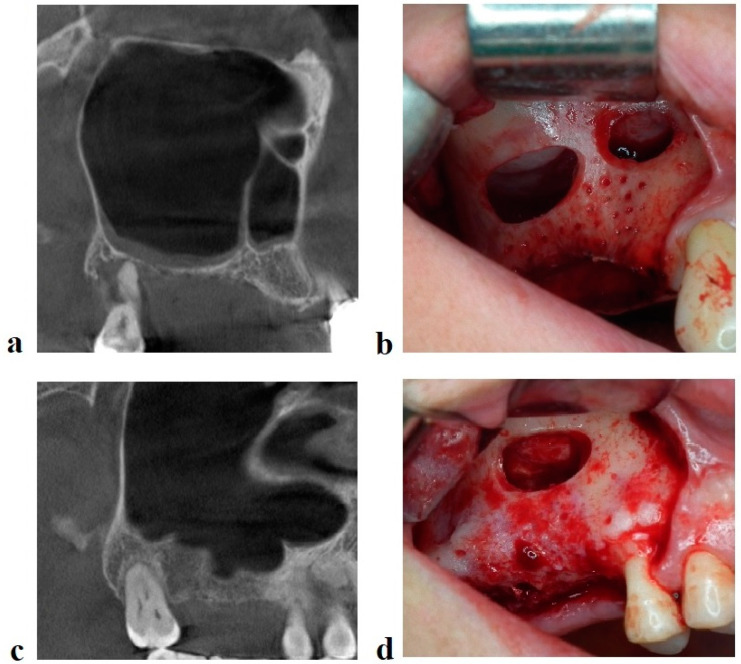
Radiological and clinical aspect of the maxillary sinus in partial edentulous patients before sinus floor augmentation. (**a**) CBCT sagittal view of a right maxilla with a long medio−lateral oriented septum. (**b**) Intraoperative view of the two windows technique. (**c**) CBCT sagittal view of a right maxilla with short medio−lateral oriented septa. (**d**) Intraoperative view of the osteotomy window.

The number of sinus septa was counted, and they were further analyzed in terms of their location, orientation, and height. When present, the antrum septa were grouped anterior, or, posterior to the zygomatic process of the maxillary bone. The anatomic landmark was the anterior limit of the zygomatic arch analyzed in the axial plane. From the point of view of the orientation, they were divided into medio-lateral and antero-posterior oriented septa. The medio-lateral type, oriented in the bucco-palatal direction, connects the buccal and palatal floors [12]. The antero-posterior type is oriented parallel to the sagittal plane. Sagittal images were used to determine the height of the medio-lateral septa [13] and the coronal images were used to determine the height of the antero-posterior septa. The examiners grouped the septa in two sizes, short ≤ 6 mm and long > 6 mm (Figure 1).

### 2.4. Statistical Analysis

The obtained data and assigned scores were recorded in a Microsoft Excel 2019 Pro Plus worksheet. Statistical analysis was performed with TIBCO Statistica 14.0.0 software (Palo Alto, CA, USA). The incidence of the septa present was documented with respect to the number of scans, the number of patients, sex of patients, sides of the maxillary sinus, and type of edentulism taking into consideration Wen’s proposed sinus septum classification.

## 3. Results

The study included 1192 maxillary sinuses from 686 patients (368 female and 318 male patients) with a mean age of 42.74 years (range between 18–73 years). They all underwent CBCT scans for different purposes (orthodontics, sinus lift procedures, third molar impaction, maxillary sinus pathologies, implant insertion, etc.). Of the entire group of patients, 506 patients had bimaxillary investigations (260 women and 246 men) and 180 had unilateral CBCTs (108 women and 72 men).

Sinus septa were detected in 504 sinuses (42.28%), while 688 sinuses (57.72%) had no internal bone crests. A total number of 652 septa were found in 504 sinuses.

The antrum septa were analyzed regarding their size, location, orientation, and number in dentate (group A), single (group B), partial (group C), and total edentulism (group D) (Table 1).

The most common orientation of the septa was medio-lateral for a number of 555 septa (85.12%), whereas the incidence of antero-posterior oriented septa was 97 (14.88%). A greater number were located posterior to the zygomatic process of the maxillary bone 409 (62.73%), while the rest were located anterior to the zygomatic process 243 (37.27%).

Regarding their size, 464 (71.17%) belonged to short category of septa (≤6 mm) and 188 (28.83%) were defined as long septa (>6 mm).

Out of 504 sinuses, 111 (22.03%) were classified as E (Easy), 234 (46.42%) as M (Moderate), and 159 (31.55%) as D (Difficult) according to Wen’s classification (Table 2).

No statistically significant correlation was observed between the right side and the left side of the same patient. Out of 506 patients with bimaxillary investigations, 298 (58.89%) belonged to the same classification level with both maxillary sinuses (ex: if the right sinus was easy, so was the left one). A number of 71 patients (14.03%) had a difference of one level in the classification (ex: if the right sinus was easy, the left one was medium, or, if the right sinus was medium, the left one was difficult), 88 patients (17.39%) had a difference of two levels in the classification (ex: if the right sinus was easy, the left one was difficult) and 49 patients (9.69%) had no septum on one side and difficult septa on the other side.

Inferential statistics were used to analyze data collected from 1192 maxillary sinus investigations. Pearson’s chi-squared test showed that Wen class MB is significantly more likely to be found on the right side (*p* = 0.0006), and that class DC is more likely to be found on the left side of the maxilla (*p* = 0.0002). Long septa are more likely to be found on the right side of the maxilla (*p* = 0.033) while short septa are on the left side (*p* = 0.022). Single septa are more likely to be found on the right side of the maxilla (*p* = 0.0002) while 2 septa are on the left side (*p* < 0.0001).

No correlation was observed between the presence of septa and the type of edentulism.

Multivariate analysis showed no statistically significant correlation between the surgery’s complexity (easy to difficult) and the patient’s sex, type of edentulism, and left or right side of the maxilla. 

## 4. Discussion

Sinus floor augmentation is often a simple, safe, and viable technique for pre-implant site augmentation surgeries. This is also one of the most frequent bone grafting procedures used for bone atrophy in the posterior region of the upper arch. The guarantee of a qualitative bone volume in the implant site represents a prerequisite for long time implant survival. Successful osseointegration of the bone substitute material and the implants depends on the accuracy of each treatment stage, from preoperative planning to postoperative protocol. The surgery may involve a variety of immediate, intraoperative, or delayed, postoperative complications, which can impede proper consolidation of the bone grafting material, or even bring additional damage to an already atrophied implant site.

The most frequent intraoperative complication of sinus floor elevation surgeries is the Schneiderian membrane perforation [14,15]. There is a large spectrum of possible causes for the sinus membrane perforation cited in the literature, such as the presence of the antrum septa, a frail Schneiderian membrane, thicker lateral walls, lack of experience of the surgeon, incorrect, abusive use of the instruments during antrostomy or during elevation [11,14,16,17,18,19,20,21,22,23,24].

To minimize the risk of complications of maxillary sinus floor elevation and other surgeries in this region, it is crucial to be familiar with different anatomic and pathologic findings in the sinus [25,26,27]. As the maxillary sinuses are significant anatomic structures in dentistry, their accurate radiological assessment is necessary, and considering CBCT as an important diagnostic method in dentistry, the recognition of anatomic variations of the maxillary sinuses in CBCT is noteworthy [25,28].

The antrum septa are extensions of the cortical plate, arising from the sinus floor, or from the walls, which can divide the maxillary sinus into two or more smaller cavities [29]. They are different in size, location, direction, thickness, number, and origin [1,29]. For this reason, there are many classifications proposed in the literature in order to have an organized description of the sinus septa [29].

Upon the congenital, or, inherited origin they are divided into primary and secondary septa [30,31,32,33]. They can result during middle face growth, as fragments of the ethmoidal infundibulum remain non-resorbed (primary septa) [30]. Another process that leads to sinus septa formation is the bone atrophy of the subantral bone (and maxillary sinus pneumatization) secondary to tooth extraction. The result is bone projections, indentations, and crests arising from the floor of the maxillary sinus (secondary septa) [30,31,32].

The incidence of the antral septa from our study corresponds to other results found in the literature. The overall prevalence of the sinus septa reported in the literature at the maxillary sinus level rates between 29.7% and 59.7% [5,26,28,29,34,35,36,37,38,39,40,41,42,43,44,45,46,47]. The incidence of the sinus septa identified on CBCT scan is directly associated with a risk of Schneiderian membrane perforation and the subsequent complications of the sinus membrane rupture (graft migration, infection, maxillary sinusitis) [11,16,17,18,19,20,21,22,23,24]. The incidence of the Schneiderian membrane perforation associated with the presence of antrum septa was 44.7% in a study performed by Irinakis and agreed with data from previous reports [47].

Besides the number, Wen attributes more characteristics to septa found in the maxillary sinus, in order to define them as easy (E), moderate (M) or difficult (D). He combines their incidence with their location, orientation and size. He also suggests a treatment option for each clinical situation [1].

From the results of our research in correlation to our clinical experience, it is relevant to divide the subclass of Difficult septa (D) proposed by Wen, into Difficult (D) including multiple medio-lateral oriented septa and short antero-posterior septa, and Highly Difficult (HD) including the long antero-posterior oriented septa and a combination of medio-lateral and antero-posterior septa (Table 3). The rationale for this separation is the degree of complexity in sinus augmentation surgeries.

**Figure 2:**
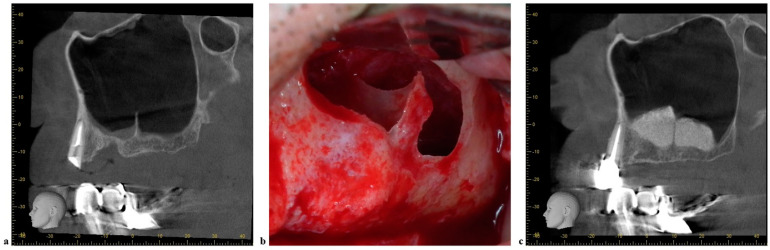
Management of medio−lateral septa. (**a**) CBCT sagittal view of the left maxilla with a medio−lateral oriented septum. (**b**) One window with a modified design of the osteotomy. (**c**) CBCT sagittal view after maxillary sinus floor augmentation.

Single or multiple medio-lateral oriented septa may still offer the possibility to elevate the membrane with a reduced risk of perforation compared to antero-posterior oriented septa. The surgeon can perform a different design of the osteotomy window to have full access on both sides of the septa during sinus membrane elevation. Multiple windows, or a single window with a wall-off technique and a modified design, can offer the possibility to elevate the sinus membrane from the anterior and the posterior sides of a medio-lateral oriented septa (Figure 2).

Antero-posterior oriented septa impede proper access and reduce partially or totally the operator’s visibility on the medial side, which increases the risk of membrane perforation.

In cases with short antero-posterior oriented septa, the Schneiderian membrane can be elevated on the lateral side, from the base to the upper edge, without risk of perforation. Depending on each clinical case (membrane thickness, inclination of the septa) the membrane can be elevated partially or totally from the medial side of the short septa.

For long septa with antero-posterior orientation, Wen proposed a crestal approach, which increases the visibility of the operator. The window preparation has to be made in a way that its border is beyond the extent of the septum anteroposteriorly. After removing the window wall, the septum is separated from the alveolar ridge and the membrane is elevated to the planned height [1]. This type of surgical intervention is more difficult than a lateral approach and requires a skilled surgeon.

**Figure 3:**
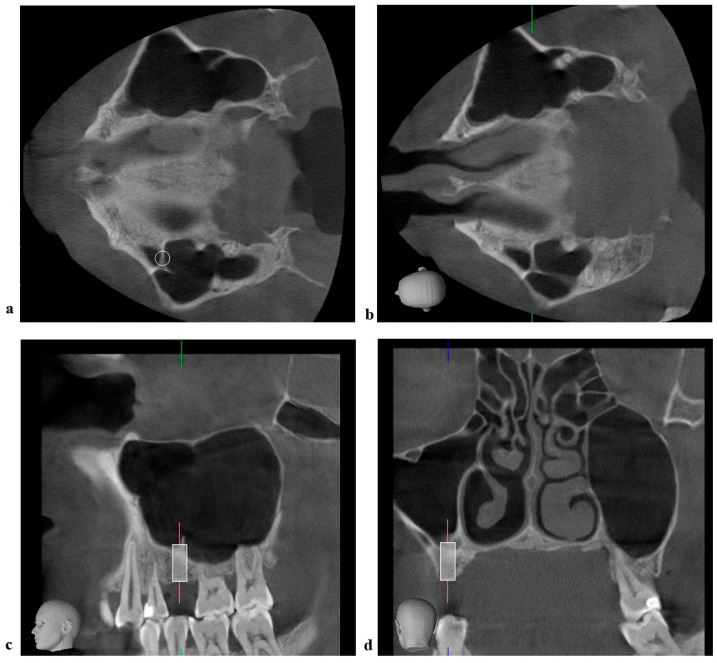
Combination of medio−lateral and antero−posterior septa (“Y” septum). (**a**) CBCT axial view of the left maxilla with simulation of implant placement. (**b**) CBCT axial view of the left maxilla revealing the “Y” septum. (**c**) CBCT sagittal view of the left maxilla with simulation of implant placement. (**d**) CBCT coronal view of the left maxilla with simulation of implant placement.

Combined medio-lateral and antero-posterior septa, with a direct interest in the implant site, can require a two-staged surgery: the first one to remove the septa (in case of proper perforation sealing of the membrane, this situation can turn into one staged surgery), and a second one for proper maxillary sinus floor augmentation [48]. This clinical situation also requires an experienced surgeon (Figure 3). We could not place this last type of septum in any of the Wen classes, which is why we suggest a minor modification to the initial classification (Table 3).

We consider Wen’s classification to be of major importance because it also gives treatment suggestions. These represent a useful tool for less experienced surgeons, by helping them plan the intervention properly. Every case is unique, and you should treat it accordingly, but it gives you a good starting point.

**Figure 4:**
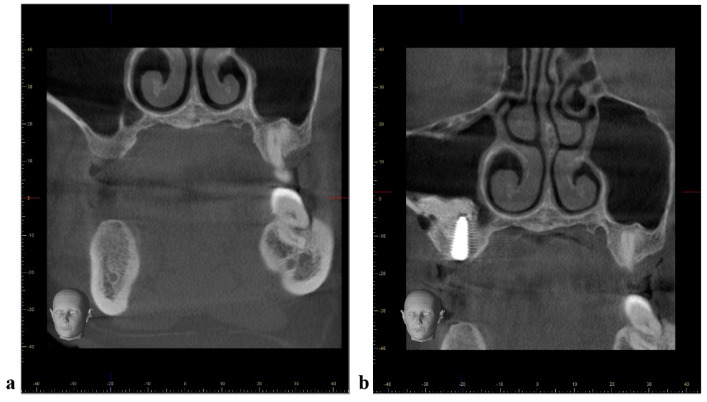
Management of antero-posterior septa. (**a**) CBCT coronal view of the left maxilla with a antero-posterior oriented septa. (**b**) CBCT coronal view after maxillary sinus floor augmentation with simultaneous dental implant placement (one window from lateral approach)—the implant prosthetically driven position did not require augmentation of the medial compartment of the sinus.

An initial planning of the implant position is relevant to know the exact surgical site which needs augmentation. In some cases, the membrane elevation and graft placement are not necessary for the entire sinus floor (Figure 4). It is our opinion that the best treatment option is the one that requires less intraoperative risks, without compromising the final outcome.

Besides the surgeon’s experience, a proper choice of surgical instruments can reduce the risk of sinus membrane ruptures. Osteotomy with a piezoelectric device to minimize trauma and intraoperative complications is highly recommended [49]. Jung et al. suggested creating a single small window anterior to the septum, or extended distally, to include the septum. After the Schneiderian membrane is lifted carefully on all sides except at the septum, a linear indentation can be made with a piezoelectric instrument at the base of the septum. Mobilization of the septum is achieved by gentle malleting and the membrane is again carefully lifted up behind the septum [50].

There are studies that show that computer guided sinus floor elevation showed promising results in accurately modifying the lateral window osteotomy and represents a safe alternative to the standard technique [51]. We should also take into consideration computer-guided sinus approach based on a magnetic stackable surgical guide to transfer the exact position of the septum and optimize the positioning of the lateral access windows. This technique reduces the risk of sinus membrane injury, thereby increasing the safety and efficacy of the procedure as Teixeira et al. previously recommended [52].

We consider the limitations of our study to be the following: the radiological exams were performed by two different cone beam computed tomography apparatuses; image processing, as well as the measurements, were performed with different software by two examiners; the uneven sample distribution of dental status. Another limitation is the lack of a correlation between the presence of sinus septa and sinus pathology (maxillary sinusitis, cystic lesions, tumors, traumatic injury, allergic sinusitis, etc.). Future studies will be required to determine more about this topic.

Within the limitations of this study, our research revealed that sinus septa are common findings, representing 42.28% in 1192 CBCT exams of the maxilla. Out of the 504 sinuses presenting septa, 111 (22.03%) were classified as E (Easy), 234 (46.42%) as M (Moderate) and 159 (31.55%) as D (Difficult) according to Wen’s classification. The most frequent subclass was MA (Moderate A) 31.94% followed by DC (Difficult C) 21.03%. 

No statistically significant correlation was observed between the surgery’s complexity (easy to difficult) and the patient’s sex, type of edentulism and left or right side of the maxilla.

## 5. Conclusions

We suggest a minor modification to Wen’s classification in view of the fact that our findings revealed a combination of medio-lateral and antero-posterior septa that we could not classify in one of the existing categories.

## Figures and Tables

**Table 1:** The incidence of sinus septa in different type of edentulism.

	No. Sinuses	Incidence of Septa
Completely dentate patients (group A)	192	38.54%
Single edentulism (group B)	320	47.19%
Partial edentulism (group C)	596	42.62%
Total edentulism (group D)	84	32.14%

**Table 2:** The incidence of surgery complexity according to Wen’s classification.

Wen Sinus Septum Classification	Number of Sinuses	Percentage
Easy/A	77	15.28%
Easy/B	34	6.75%
Medium/A	161	31.94%
Medium/B	73	14.48%
Difficult/A	35	6.95%
Difficult/B	18	3.57%
Difficult/C	106	21.03%

**Table 3:** Minor modification brought to Wen’s classification, with the combination of medio-lateral and antero-posterior septa in Highly Difficult (HD) group. In both classes (D-Difficult and HD-Highly Difficult) there are two subdivisions (A and B), which vary in different orientation of the septa and treatment options.

Classification	Subclass	Location	Number	Orientation	Size (mm)	Proposed Treatment Approach
(D) Difficult	A	Anterior or posterior to zygomatic process	≥2	Medio-lateral		Multiple windows and/or wall-gone technique
	B		1	Antero-posterior	≤6	One window from the lateral approach
Highly Difficult (HD)	A	Anterior or posterior to zygomatic process	1	Antero-posterior	>6	One window from the crestal approach & the wall gone technique
	B			Combination of medio-lateral and antero-posterior septa		One window from the lateral approach and removal of the septum; most likely second surgery for sinus floor augmentation

## Data Availability

The data presented in this study are available on request from the corresponding author. The data are not publicly available due to privacy policy.

## References

[1] Wen S.C., Chan H.L., Wang H.L. (2013). Classification and management of antral septa for maxillary sinus augmentation. Int. J. Periodontics Restor. Dent..

[2] Aşantoğrol F., Coşgunarslan A. (2021). The effect of anatomical variations of the sinonasal region on maxillary sinus volume and dimensions: A three-dimensional study. Braz. J. Otorhinolaryngol..

[3] Whyte A., Boeddinghaus R. (2019). The maxillary sinus: Physiology, development and imaging anatomy. Dentomaxillo Facial Radiol..

[4] Malec M., Smektała T., Trybek G., Sporniak-Tutak K. (2013). Maxillary sinus septa: Prevalence, morphology, diagnostics and implantological implications. Systematic review. Folia Morphol..

[5] Tadinada A., Jalali E., Al-Salman W., Jambhekar S., Katechia B., Almas K. (2016). Prevalence of bony septa, antral pathology, and dimensions of the maxillary sinus from a sinus augmentation perspective: A retrospective cone-beam computed tomography study. Imaging Sci. Dent..

[6] Reda R., Zanza A., Mazzoni A., Cicconetti A., Testarelli L., Di Nardo D. (2021). An Update of the Possible Applications of Magnetic Resonance Imaging (MRI) in Dentistry: A Literature Review. J. Imaging.

[7] Patil S., Alkahtani A., Bhandi S., Mashyakhy M., Alvarez M., Alroomy R., Hendi A., Varadarajan S., Reda R., Raj A.T. (2021). Ultrasound Imaging versus Radiographs in Differentiating Periapical Lesions: A Systematic Review. Diagnostics.

[8] Hsu C.C., Sheng C., Ho C.Y. (2018). Efficacy of sinus ultrasound in diagnosis of acute and subacute maxillary sinusitis. J. Chin. Med. Assoc..

[9] Shokri A., Jamalpour M., Jafariyeh B., Poorolajal J., Sabet N.K. (2017). Comparison of Ultrasonography, Magnetic Resonance Imaging and Cone Beam Computed Tomography for Detection of Foreign Bodies in Maxillofacial Region. J. Clin. Diagn. Res..

[10] Okuyama K., Sakamoto Y., Naruse T., Kawakita A., Yanamoto S., Furukawa K., Umeda M. (2017). Intraoral extraction of an ectopic mandibular third molar detected in the subcondylar region without a pathological cause: A case report and literature review. CRANIO®—J. Craniomandib. Sleep Pract..

[11] Schwarz L., Schiebel V., Hof M., Ulm C., Watzek G., Pommer B. (2015). Risk Factors of Membrane Perforation and Postoperative Complications in Sinus Floor Elevation Surgery: Review of 407 Augmentation Procedures. J. Oral Maxillofac. Surg..

[12] Qian L., Tian X.M., Zeng L., Gong Y., Wei B. (2016). Analysis of the Morphology of Maxillary Sinus Septa on Reconstructed Cone-Beam Computed Tomography Images. J. Oral Maxillofac. Surg..

[13] Jang S.Y., Chung K., Jung S., Park H.J., Oh H.K., Kook M.S. (2014). Comparative study of the sinus septa between dentulous and edentulous patients by cone beam computed tomography. Implant Dent..

[14] Barbu H.M., Iancu S.A., Jarjour Mirea I., Mignogna M.D., Samet N., Calvo-Guirado J.L. (2019). Management of Schneiderian Membrane Perforations during Sinus Augmentation Procedures: A Preliminary Comparison of Two Different Approaches. J. Clin. Med..

[15] Bathla S.C., Fry R.R., Majumdar K. (2018). Maxillary sinus augmentation. J. Indian Soc. Periodontol..

[16] Stacchi C., Lombardi T., Cusimano P., Berton F., Lauritano F., Cervino G., Di Lenarda R., Cicciù M. (2017). Bone Scrapers Versus Piezoelectric Surgery in the Lateral Antrostomy for Sinus Floor Elevation. J. Craniofac. Surg..

[17] Al-Dajani M. (2016). Incidence, Risk Factors, and Complications of Schneiderian Membrane Perforation in Sinus Lift Surgery: A Meta-Analysis. Implant Dent..

[18] von Arx T., Fodich I., Bornstein M.M., Jensen S.S. (2014). Perforation of the sinus membrane during sinus floor elevation: A retrospective study of frequency and possible risk factors. Int. J. Oral Maxillofac. Implant..

[19] Sigaroudi A.K., Kajan Z.D., Rastgar S., Asli H.N. (2017). Frequency of different maxillary sinus septal patterns found on cone-beam computed tomography and predicting the associated risk of sinus membrane perforation during sinus lifting. Imaging Sci. Dent..

[20] Tükel H.C., Tatli U. (2018). Risk factors and clinical outcomes of sinus membrane perforation during lateral window sinus lifting: Analysis of 120 patients. Int. J. Oral Maxillofac. Surg..

[21] Marin S., Kirnbauer B., Rugani P., Payer M., Jakse N. (2019). Potential risk factors for maxillary sinus membrane perforation and treatment outcome analysis. Clin. Implant Dent. Relat. Res..

[22] Krennmair S., Malek M., Forstner T., Krennmair G., Weinländer M., Hunger S. (2020). Risk Factor Analysis Affecting Sinus Membrane Perforation During Lateral Window Maxillary Sinus Elevation Surgery. Int. J. Oral Maxillofac. Implant..

[23] Pizzini A., Basma H.S., Li P., Geurs N.C., Abou-Arraj R.V. (2021). The impact of anatomic, patient and surgical factors on membrane perforation during lateral wall sinus floor elevation. Clin. Oral Implants Res..

[24] Testori T., Yu S.H., Tavelli L., Wang H.L. (2020). Perforation Risk Assessment in Maxillary Sinus Augmentation with Lateral Wall Technique. Int. J. Periodontics Restor. Dent..

[25] Shahidi S., Zamiri B., Danaei S.M., Salehi S., Hamedani S. (2016). Evaluation of Anatomic Variations in Maxillary Sinus with the Aid of Cone Beam Computed Tomography (CBCT) in a Population in South of Iran. J. Dent..

[26] Orhan K., Seker B.K., Aksoy S., Bayindir H., Berberoğlu A., Seker E. (2013). Cone beam CT evaluation of maxillary sinus septa prevalence, height, location and morphology in children and an adult population. Med. Princ. Pract..

[27] Dobele I., Kise L., Apse P., Kragis G., Bigestans A. (2013). Radiographic assessment of findings in the maxillary sinus using cone-beam computed tomography. Stomatologija.

[28] Lana J.P., Carneiro P.M., Machado V., de Souza P.E., Manzi F.R., Horta M.C. (2012). Anatomic variations and lesions of the maxillary sinus detected in cone beam computed tomography for dental implants. Clin. Oral Implant. Res..

[29] Nasseh I., Aoun G., El-Outa A., Nassar J., Nasseh P., Hayek E. (2020). Mapping Maxillary Sinus Septa in a Lebanese Sample: A Radio-anatomical Study. Acta Inform. Med..

[30] Faramarzie M., Babaloo A.R., Oskouei S.G. (2009). Prevalence, height, and location of antral septa in Iranian patients undergoing maxillary sinus lift. J. Adv. Periodontol. Implant. Dent..

[31] Demirkol M., Demirkol N. (2019). The effects of posterior alveolar bone height on the height of maxillary sinus septa. Surg. Radiol. Anat..

[32] Bornstein M.M., Seiffert C., Maestre-Ferrín L., Fodich I., Jacobs R., Buser D., von Arx T. (2016). An Analysis of Frequency, Morphology, and Locations of Maxillary Sinus Septa Using Cone Beam Computed Tomography. Int. J. Oral Maxillofac. Implant..

[33] Al-Zahrani M.S., Al-Ahmari M.M., Al-Zahrani A.A., Al-Mutairi K.D., Zawawi K.H. (2020). Prevalence and morphological variations of maxillary sinus septa in different age groups: A CBCT analysis. Ann. Saudi Med..

[34] Yildirim T.T., Güncü G.N., Colak M., Nares S., Tözüm T.F. (2017). Evaluation of maxillary sinus septa: A retrospective clinical study with cone beam computerized tomography (CBCT). Eur. Rev. Med. Pharmacol. Sci..

[35] Schriber M., von Arx T., Sendi P., Jacobs R., Suter V.G., Bornstein M.M. (2017). Evaluating Maxillary Sinus Septa Using Cone Beam Computed Tomography: Is There a Difference in Frequency and Type Between the Dentate and Edentulous Posterior Maxilla?. Int. J. Oral Maxillofac. Implant..

[36] Takeda D., Hasegawa T., Saito I., Arimoto S., Akashi M., Komori T. (2019). A radiologic evaluation of the incidence and morphology of maxillary sinus septa in Japanese dentate maxillae. Oral Maxillofac. Surg..

[37] Jung J., Park J.S., Hong S.J., Kim G.T., Kwon Y.D. (2020). Axial Triangle of the Maxillary Sinus, and its Surgical Implication With the Position of Maxillary Sinus Septa During Sinus Floor Elevation: A CBCT Analysis. J. Oral Implant..

[38] Toprak M.E., Ataç M.S. (2021). Maxillary sinus septa and anatomical correlation with the dentition type of sinus region: A cone beam computed tomographic study. Br. J. Oral Maxillofac. Surg..

[39] Rancitelli D., Borgonovo A.E., Cicciù M., Re D., Rizza F., Frigo A.C., Maiorana C. (2015). Maxillary Sinus Septa and Anatomic Correlation With the Schneiderian Membrane. J. Craniofac. Surg..

[40] Sakhdari S., Panjnoush M., Eyvazlou A., Niktash A. (2016). Determination of the Prevalence, Height, and Location of the Maxillary Sinus Septa Using Cone Beam Computed Tomography. Implant Dent..

[41] Chen M.Z., Xie Y.F., Xie H., Wang G.H., He J.C. (2016). Cone-beam CT study of bone septa during maxillary sinus lift among Changzhou population. Shanghai J. Stomatol..

[42] Taleghani F., Tehranchi M., Shahab S., Zohri Z. (2017). Prevalence, Location, and Size of Maxillary Sinus Septa: Computed Tomography Scan Analysis. J. Contemp. Dent. Pract..

[43] Shen E.C., Fu E., Chiu T.J., Chang V., Chiang C.Y., Tu H.P. (2012). Prevalence and location of maxillary sinus septa in the Taiwanese population and relationship to the absence of molars. Clin. Oral Implant. Res..

[44] Kang S.J., Shin S.I., Herr Y., Kwon Y.H., Kim G.T., Chung J.H. (2013). Anatomical structures in the maxillary sinus related to lateral sinus elevation: A cone beam computed tomographic analysis. Clin. Oral Implant. Res..

[45] Li J., Zhou Z.X., Yuan Z.Y., Yuan H., Sun C., Chen N. (2013). An anatomical study of maxillary sinus septum of Han population in Jiangsu region using cone-beam CT. Shanghai J. Stomatol..

[46] Hungerbühler A., Rostetter C., Lübbers H.T., Rücker M., Stadlinger B. (2019). Anatomical characteristics of maxillary sinus septa visualized by cone beam computed tomography. Int. J. Oral Maxillofac. Surg..

[47] Irinakis T., Dabuleanu V., Aldahlawi S. (2017). Complications During Maxillary Sinus Augmentation Associated with Interfering Septa: A New Classification of Septa. Open Dent. J..

[48] Okada T., Kawana H. (2019). Two-Step Procedure for the Treatment of a Maxillary Sinus with Complex Sinus Septa: A Highly Predictive Method for Sinus Floor Augmentation After Perforation of the Maxillary Sinus Membrane. Int. J. Periodontics Restor. Dent..

[49] Martins M., Vieira W.A., Paranhos L.R., Motta R.H., da Silva C.S., Rodriguez C., Ramacciato J.C. (2021). Comparison of piezosurgery and conventional rotary instruments in schneider’s membrane sinus lifting: A pilot randomized trial. J. Clin. Exp. Dent..

[50] Jung J., Hwang B.Y., Kim B.S., Lee J.W. (2019). Floating septum technique: Easy and safe method maxillary sinus septa in sinus lifting procedure. Maxillofac. Plast. Reconstr. Surg..

[51] Osman A.H., Mansour H., Atef M., Hakam M. (2018). Computer guided sinus floor elevation through lateral window approach with simultaneous implant placement. Clin. Implant Dent. Relat. Res..

[52] Teixeira K.N., Sakurada M.A., Philippi A.G., Gonçalves T. (2021). Use of a stackable surgical guide to improve the accuracy of the lateral wall approach for sinus grafting in the presence of a sinus septum. Int. J. Oral Maxillofac. Surg..

